# Medial femoral condyle bone mineral density is associated with subchondral insufficiency fractures in female patients with medial meniscus posterior root tears

**DOI:** 10.1002/jeo2.70877

**Published:** 2026-08-03

**Authors:** Koki Kawada, Kazuhisa Sugiu, Takaaki Hiranaka, Yuta Morinobu, Yuki Okazaki, Takayuki Furumatsu

**Affiliations:** ^1^ Department of Orthopaedic Surgery Okayama University Graduate School of Medicine, Dentistry, and Pharmaceutical Sciences Okayama Japan; ^2^ Department of Orthopaedic Surgery Japanese Red Cross Okayama Hospital Okayama Japan

**Keywords:** bone mineral density, meniscus, osteoporosis, posterior root tear, subchondral insufficiency fracture

## Abstract

**Purpose:**

In this study, we aimed to evaluate the correlation between bone mineral density (BMD) of the medial femoral condyle (MFC) in patients with medial meniscus posterior root tears (MMPRT) and BMD at commonly evaluated sites, including the femur and lumbar spine. We also examined the association between MFC BMD and subchondral insufficiency fractures of the knee (SIFK).

**Methods:**

This retrospective cross‐sectional study included patients diagnosed with MMPRT based on magnetic resonance imaging findings at our hospital between November 2025 and March 2026. Lower limb alignment, bone morphology, medial meniscus extrusion (MME) and SIFK grade were evaluated. BMD was measured at the proximal femur, lumbar spine and MFC using dual‐energy X‐ray absorptiometry. Spearman's rank correlation coefficient was used to evaluate the correlation between the MFC BMD and other BMD measurements. Furthermore, linear regression analysis was conducted to identify factors associated with MFC BMD.

**Results:**

A total of 47 patients were included in the final analysis. The mean age was 66.7 ± 8.2 years, and the mean body mass index (BMI) was 24.1 ± 3.9 kg/m^2^. The Kellgren–Lawrence grade distribution (0/1/2/3/4) was 0/29/18/0/0. The mean MME was 3.5 ± 1.1 mm, and the SIFK grade distribution (0/1/2/3/4) was 37/6/4/0/0. MFC BMD showed a significant positive correlation with BMD at all other measured regions (*p* < 0.001). Multivariate linear regression analysis showed that age (*β* = −0.009, *p* < 0.001), BMI (*β* = 0.009, *p* = 0.045) and the presence of SIFK in MFC (*β* = −0.129, *p* = 0.003) were each independently associated with MFC BMD.

**Conclusions:**

MFC BMD was significantly correlated with BMD at commonly assessed regions of the proximal femur and lumbar spine in patients with early‐stage osteoarthritis. Additionally, the lower MFC BMD was associated with the presence of SIFK in female patients with MMPRT.

**Level of Evidence:**

Level IV.

## Graphical abstract

Bone mineral density (BMD) of the medial femoral condyle (MFC) was significantly correlated BMD at the proximal femur and lumbar spine in female patients with medial meniscus posterior root tears (MMPRT). Lower MFC BMD was independently associated with the presence of subchondral insufficiency fractures of the knee (SIFK), suggesting that local bone fragility may contribute to SIFK development.

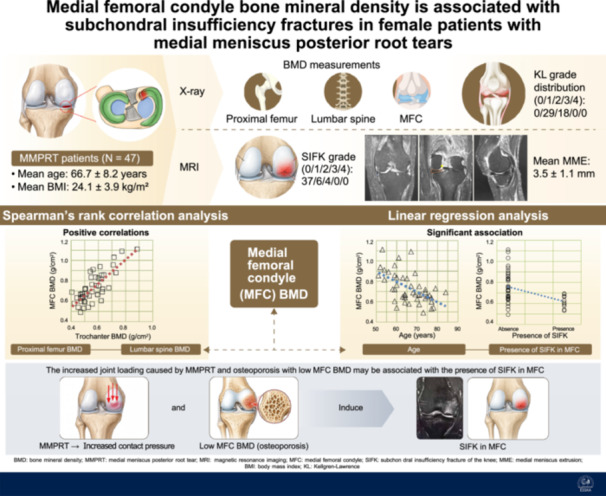

**Abbreviations**
BMD: bone mineral density
BMI: body mass index
DEXA: dual‐energy X‐ray absorptiometry
KL: Kellgren–Lawrence grade
MFC: medial femoral condyle
MME: medial meniscus extrusion
MMPRT: medial meniscus posterior root tears
MRI: magnetic resonance imaging
SIFK: subchondral insufficiency fractures of the knee

## INTRODUCTION

Subchondral insufficiency fractures of the knee (SIFK) result from microfractures caused by repeated excessive loading of the subchondral bone [13]. If SIFK does not heal, osteonecrosis or osteochondral collapse may occur [13]. Consequently, SIFK has been reported as a risk factor for the progression of osteoarthritis and for the need for surgical interventions, including joint replacement [2, 14]. Associations between SIFK and meniscal tears or medial meniscus extrusion (MME) have been reported [6, 9, 17]. In particular, several reports have suggested a potential link between SIFK and medial meniscus posterior root tears (MMPRT) [3, 4, 21]. Garcia et al. reported SIFK expression in 90 of 153 patients with isolated MMPRT [5]. However, although SIFK is frequently observed in patients with MMPRT, it did not occur in all cases.

As SIFK represents a type of insufficiency fracture, reduced bone mineral density (BMD) is considered a potential risk factor for its development. Several studies have reported an association between lower BMD and SIFK [1, 23]. However, these studies measured BMD at the distal radius or tibia. In contrast, studies evaluating BMD at commonly used sites, including the lumbar spine and proximal femur, have reported no significant association with SIFK [9, 12]. Therefore, the relationship between BMD and SIFK remains controversial. Moreover, limited evidence is available regarding the association between local BMD of the medial femoral condyle (MFC) and SIFK. Clarifying this relationship may help identify patients at increased risk of SIFK and guide treatment decisions for patients with MMPRT.

Therefore, we aimed to evaluate the association between MFC BMD and patient characteristics, imaging findings and the presence of SIFK in patients with MMPRT. The hypothesis of this study was that MFC BMD would correlate with age and the presence of SIFK.

## MATERIALS AND METHODS

### Patients

This retrospective cross‐sectional study was conducted in accordance with the Declaration of Helsinki and approved by our institutional review board. This study was reported in accordance with the Strengthening the Reporting of Observational Studies in Epidemiology (STROBE) guidelines for cross‐sectional studies [20]. Written informed consent was obtained from all patients.

**Figure 1:**
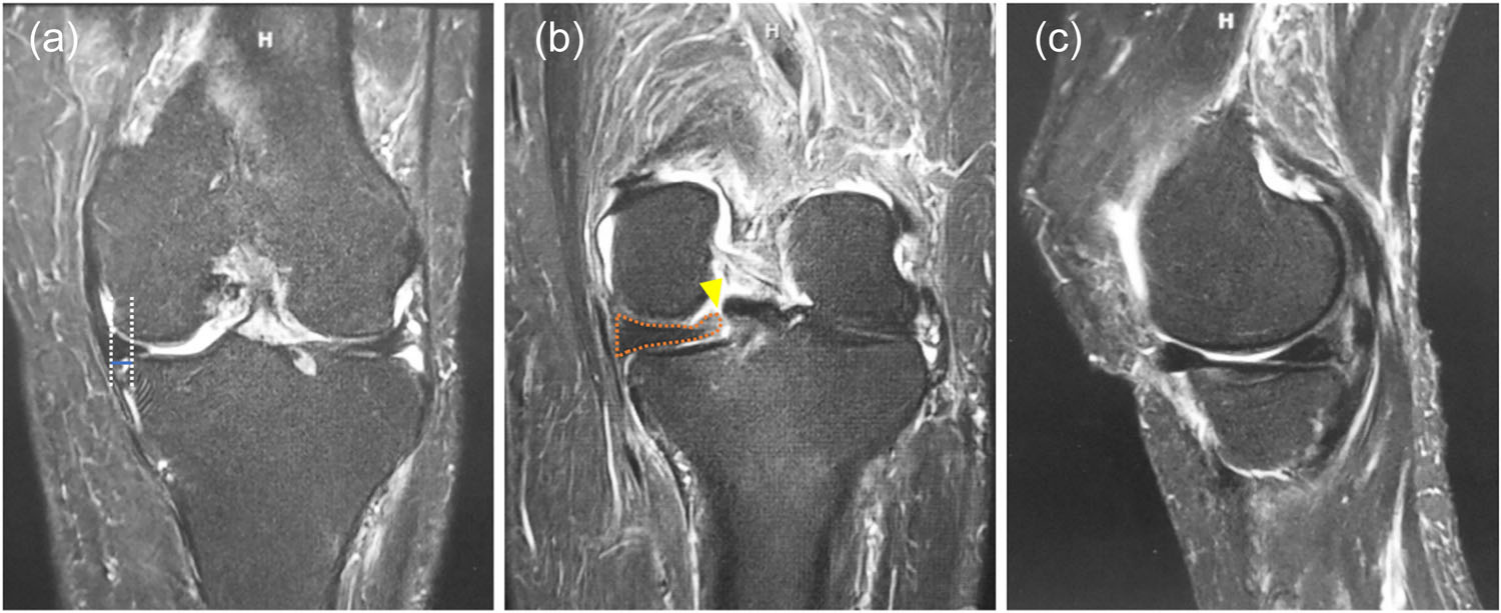
Patient characteristics and imaging findings of a female patient cohort diagnosed with MMPRT on MRI. (a) MME (blue line) is observed. No bone marrow lesion is detected in the MFC and MTP (coronal images). (b) Giraffe neck sign (orange dotted line) and cleft sign (yellow arrowhead) are observed. (c) No bone marrow lesion is observed in the medial compartment (sagittal images). MFC, medial femoral condyle; MME, medial meniscus extrusion; MMPRT, medial meniscus posterior root tears; MRI, magnetic resonance imaging; MTP, medial tibial plateau; SIFK, subchondral insufficiency fractures of the knee.

This study included patients diagnosed with MMPRT based on magnetic resonance imaging (MRI) findings at our hospital between November 2025 and March 2026 (Figure 1). The exclusion criteria were male sex and age < 50 years. No patients had a history of prior surgery on the MMPRT‐affected knee or any inflammatory disease.

At our institution, transtibial pullout repair is generally recommended for patients with symptomatic MMPRT who have a Kellgren–Lawrence (KL) grade ≤ 2, femorotibial angle ≤ 180°, cartilage lesions of modified outerbridge grade ≤ 2 or focal modified outerbridge grade > 2 lesions measuring ≤1.5 cm^2^ [16] and no advanced subchondral involvement (SIFK grade ≤ 2) [17]. Patients who do not meet these criteria, or who choose conservative management, are treated nonoperatively. For those with persistent symptoms despite nonoperative treatment, options such as around‐knee osteotomy or knee arthroplasty may be considered.

### Radiological assessments

The radiographic images obtained included standing anteroposterior knee radiographs, Rosenberg views and non‐weight‐bearing lateral knee radiographs. Using standing anteroposterior knee radiographs, the femorotibial and medial proximal tibial angles were measured [7, 11]. Using the Rosenberg view, the KL grade was assessed [15]. Using lateral knee radiographs, in which the medial and lateral posterior femoral condyles were aligned, the medial posterior tibial slope angle was assessed [19].

### MRI assessments

MRI of the knee joint was performed in the non‐weight‐bearing position. MME was evaluated using coronal MRI. MME was defined as the distance from the medial margin of the tibial plateau (excluding osteophytes) to the medial margin of the medial meniscus, measured on the slice showing highest medial tibial eminence [8]. SIFK grading in the MFC was also assessed using MRI [17]. The grading system was defined as follows: Grade 1, contusion of the subchondral plate without a subchondral fracture; Grade 2, presence of a subchondral fracture without subchondral cystic changes or osteonecrosis; Grade 3, presence of subchondral cystic changes without osteonecrosis; and Grade 4, presence of osteonecrosis and/or subchondral collapse.

### BMD assessments

**Figure 2:**
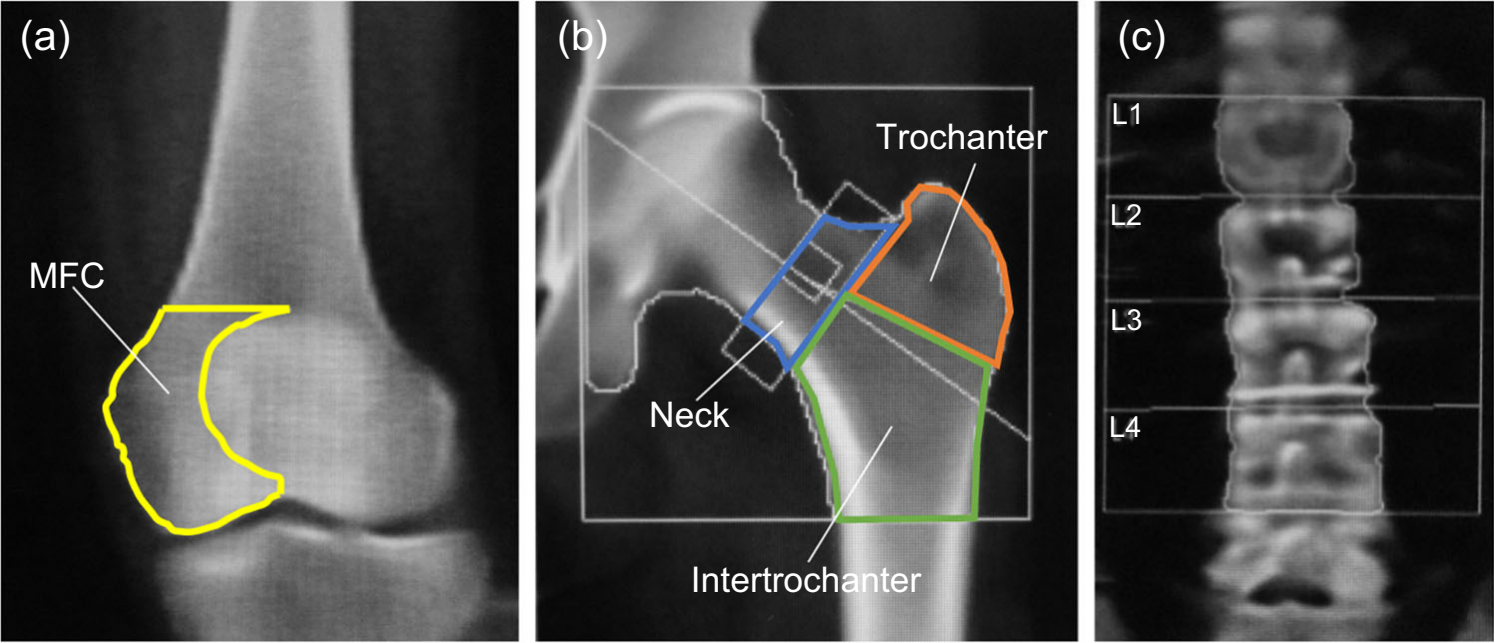
BMD measurement of each skeletal region using DEXA in female patients diagnosed with MMPRT on MRI. (a) BMD of the MFC (yellow line) was measured by defining a region from the superior border of the patella to the MFC, excluding the overlapping portion of the patella. (b) BMD of the proximal femur was measured at the femoral neck (blue line), trochanteric region (orange line), intertrochanteric region (green line) and the total hip region. (c) BMD of the lumbar spine was measured from L1 to L4. BMD, bone mineral density; DEXA, dual‐energy X‐ray absorptiometry; MFC, medial femoral condyle; MMPRT, medial meniscus posterior root tears; MRI, magnetic resonance imaging.

BMD was measured using a dual‐energy X‐ray absorptiometry (DEXA) system (Horizon Wi; Hologic Inc.) with software version 13.6.1.3. This system allows whole‐body BMD assessment, and flexible region‐of‐interest settings enable detailed local analysis. The BMD of the MFC was measured by defining a region extending from the superior border of the patella to the MFC, excluding the overlapping portion of the patella (Figure 2a). BMD of the proximal femur was measured at the femoral neck, trochanteric region, intertrochanteric region and total hip (Figure 2b). BMD of the lumbar spine was measured from L1 to L4 (Figure 2c).

### Statistical analysis

Statistical analyses were performed using EZR software (Saitama Medical Center). The Shapiro–Wilk normality test was used to assess the normality of the distribution.

Spearman's rank correlation analysis was used to evaluate the correlations between MFC BMD and other BMD measurements.

Univariable linear regression analyses were performed to assess the associations between patient characteristics, imaging findings and MFC BMD. Variables with a *p* value ≤ 0.200 in the univariable analyses were subsequently entered into a multivariable linear regression analysis.

An a priori power analysis for correlation analysis was performed using G*Power software (Version 3.1.9.7; Heinrich Heine University Düsseldorf). Assuming an expected correlation coefficient of 0.40, a two‐sided alpha level of 0.05 and power of 0.80, the required sample size was estimated to be 46 patients.

Imaging findings were independently assessed by two observers, and interobserver reliability was assessed using intraclass correlation coefficients (ICCs).

## RESULTS

Between November 2025 and March 2026, 61 patients were diagnosed with MMPRT based on MRI findings at our institution. Of these, 47 female patients aged ≥ 50 years were included in the final analysis after excluding 12 male patients and 2 patients younger than 50 years. Among the included patients, 44 underwent transtibial pullout repair, 2 underwent unicompartmental knee arthroplasty, and 1 received conservative treatment.

Patient characteristics and imaging findings are summarized in Table 1, and BMD measurements are summarized in Table 2.

**Table 1:** Patient characteristics and imaging findings.

	Values	Range
Patient characteristics		
Patients, *n*	47	
Age (years)	66.7 ± 8.2	51–84
Sex, male/female, *n*	0/47	
BMI (kg/m^2^)	24.1 ± 3.9	16.9–31.5
Imaging findings		
KL grade, 0/1/2/3/4, *n*	0/29/18/0/0	
Femorotibial angle (°)	178.1 ± 1.4	175–180
Medial proximal tibial angle (°)	84.9 ± 2.0	81–92
Medial posterior tibial slope (°)	9.7 ± 2.4	5–15
MME (mm)	3.5 ± 1.1	1.2–6.2
SIFK grade in MFC, 0/1/2/3/4, *n*	37/6/4/0/0	

*Note*: Values are presented as the mean ± standard deviation or number (*n*).

Abbreviations: BMI, body mass index; KL, Kellgren–Lawrence; MFC, medial femoral condyle; MME, medial meniscus extrusion; SIFK, subchondral insufficiency fractures of the knee.

**Table 2:** BMD values at each skeletal measurement region using DEXA in a female patient cohort diagnosed with MMPRT on MRI.

BMD measurement site	Values (g/cm^2^)	Range (g/cm^2^)
Medial femoral condyle	0.729 ± 0.144	0.493–1.111
Total femur	0.738 ± 0.112	0.556–1.132
Femoral neck	0.598 ± 0.095	0.456–0.951
Trochanter	0.562 ± 0.096	0.406–0.886
Intertrochanter	0.884 ± 0.140	0.645–1.371
Lumbar spine 1–4	0.851 ± 0.176	0.595–1.430

*Note*: Values are presented as the mean ± standard deviation.

Abbreviations: BMD, bone mineral density; DEXA, dual‐energy X‐ray absorptiometry; MMPRT, medial meniscus posterior root tears; MRI, magnetic resonance imaging.

**Figure 3:**
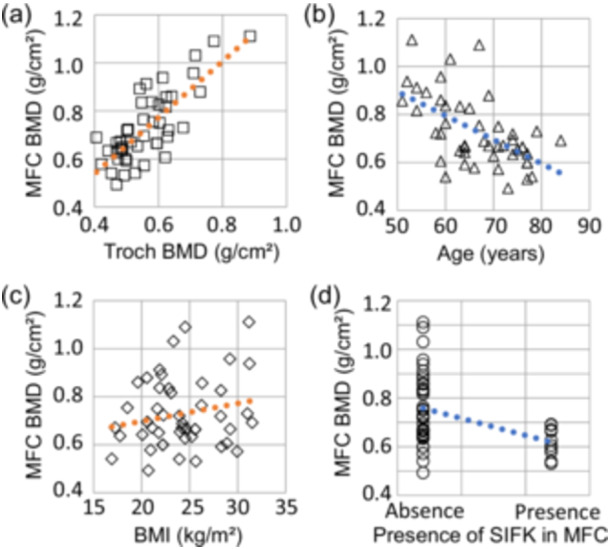
Associations between MFC BMD and related variables in a female patient cohort diagnosed with MMPRT on MRI. (a) Scatter plot showing the correlation between MFC BMD and trochanteric BMD (*r* = 0.707, *p* < 0.001). (b) Association between age and MFC BMD identified by multivariable linear regression analysis (*β* = −0.009, *p* < 0.001). (c) Association between BMI and MFC BMD identified by multivariable linear regression analysis (*β* = 0.009, *p* = 0.045). (d) Association between the presence of SIFK in the MFC and MFC BMD identified by multivariable linear regression analysis (*β* = −0.129, *p* = 0.003). BMD, bone mineral density; BMI, body mass index; MFC, medial femoral condyle; MMPRT, medial meniscus posterior root tears; MRI, magnetic resonance imaging; SIFK, subchondral insufficiency fractures of the knee; Troch, trochanter.

MFC BMD showed a significant positive correlation with BMD at all other measurement regions (*p *< 0.001; Table 3). In particular, MFC BMD and trochanter BMD showed a strong positive correlation (*r* = 0.707, *p *< 0.001; Figure 3a).

**Table 3:** Spearman's rank correlations between MFC BMD and other BMD measurement regions.

BMD measurement site	*r*	95% CI	*p* Value
Total femur	0.696	0.511–0.819	**<0.001** *
Femoral neck	0.666	0.468–0.800	**<0.001** *
Trochanter	0.707	0.527–0.826	**<0.001** *
Intertrochanter	0.675	0.484–0.806	**<0.001** *
Lumbar spine 1–4	0.651	0.452–0.792	**<0.001** *

*Note*: Bold values indicate statistical significance.

Abbreviations: BMD, bone mineral density; CI, confidence interval; MFC, medial femoral condyle.

* *p* < 0.05, statistically significant.

In univariable linear regression analyses, age (*β* = −0.010, *p* < 0.001) and the presence of SIFK in MFC (*β* = −0.139, *p *= 0.006) were significantly associated with MFC BMD (Table 4). Furthermore, in the multivariate linear regression analyses, age (*β* = −0.009, *p *< 0.001; Figure 3b), body mass index (BMI) (*β* = 0.009, *p *= 0.045; Figure 3c) and the presence of SIFK in MFC (β = −0.129, *p *= 0.003; Figure 3d) were identified as independent factors associated with MFC BMD (Table 5).

**Table 4:** Univariable linear regression analysis of factors associated with MFC BMD.

	*β* coefficient	95% CI	*p* Value
Patient characteristics			
Age	−0.010	−0.014 to −0.006	**<0.001** *
BMI	0.007	−0.004 to 0.019	0.183
Imaging findings			
KL grade	−0.051	−0.138 to 0.037	0.253
Femorotibial angle	0.008	−0.024 to 0.039	0.631
Medial proximal tibial angle	0.011	−0.011 to 0.032	0.331
Medial posterior tibial slope	−0.010	−0.028 to 0.007	0.252
MME	−0.005	−0.046 to 0.036	0.798
Presence of SIFK in MFC	−0.139	−0.237 to −0.042	**0.006** *

*Note*: Bold values indicate statistical significance.

Abbreviations: BMD, bone mineral density; BMI, body mass index; CI, confidence interval; KL, Kellgren–Lawrence; MFC, medial femoral condyle; MME, medial meniscus extrusion; SIFK, subchondral insufficiency fractures of the knee.

* *p* < 0.05, statistically significant.

**Table 5:** Multivariable linear regression analysis of factors associated with MFC BMD.

	*β* coefficient	95% CI	*p* Value
Patient characteristics			
Age	−0.009	−0.013 to −0.005	**<0.001** *
BMI	0.009	0.0002–0.018	**0.045** *
Imaging findings			
Presence of SIFK in MFC	−0.129	−0.212 to −0.047	**0.003** *

*Note*: Bold values indicate statistical significance.

Abbreviations: BMD, bone mineral density; BMI, body mass index; CI, confidence interval; MFC, medial femoral condyle; SIFK, subchondral insufficiency fractures of the knee.

* *p* < 0.05, statistically significant.

All interobserver ICCs were greater than 0.80, indicating good reliability.

## DISCUSSION

The two key findings of this study are as follows. First, MFC BMD was found to have a significant correlation with the BMD of commonly assessed regions of the proximal femur and lumbar spine. Second, lower MFC BMD was associated with older age, lower BMI and the presence of SIFK in the MFC.

BMD is primarily assessed using DEXA at the lumbar spine and proximal femur (total hip and femoral neck), which are standard and the most commonly measured regions according to the current international guidelines [18]. Therefore, BMD measurements are rarely performed in the other regions. Lee et al. reported that BMD in the proximal humerus showed no significant correlation with BMD in the proximal femur or lumbar spine and should therefore be evaluated separately [10]. Conversely, Yoon et al. reported that BMD in the around‐the‐knee regions correlates with BMD in the proximal femur and lumbar spine in patients with advanced osteoarthritis who underwent total knee arthroplasty [22]. However, in patients with advanced osteoarthritis suitable for total knee arthroplasty, it is necessary to consider the possibility that osteophytes, bone sclerosis and changes in the weight‐bearing load environment may significantly affect MFC BMD. Meanwhile, in the present study, MFC BMD showed a strong positive correlation with the BMD of the proximal femur and lumbar spine in patients with early‐stage osteoarthritis suitable for meniscal repair. In particular, the BMD of the trochanteric region showed a stronger positive correlation with MFC BMD. This finding may be attributed to similarities in bone composition, as both the MFC and trochanteric regions are predominantly composed of cancellous bone.

Regarding the relationship between BMD and SIFK, previous studies have reported no association between BMD in the proximal femur or lumbar spine and the development of SIFK [9, 12]. However, these studies were not limited to female patients or to those with MMPRT. In the present study, which included only female patients with MMPRT, MFC BMD was identified as an independent risk factor for the presence of SIFK in the MFC. As SIFK is a type of insufficiency fracture, these findings suggest that low local MFC BMD, together with increased subchondral loading due to MMPRT, may contribute to the development and severity of SIFK. Furthermore, these findings indicate that assessment of BMD may contribute to treatment decision‐making in patients with MMPRT. Patients with low BMD may require careful monitoring during conservative management or while awaiting surgery. In addition, pharmacological interventions for osteoporosis may have the potential to prevent the development of SIFK. Future prospective studies are needed to determine whether low BMD predicts the development of SIFK in patients with MMPRT and whether osteoporosis treatment reduces the risk of SIFK onset or progression.

This study has several limitations. First, this retrospective study was subject to potential selection bias and unmeasured confounding. Second, the sample size was relatively small. Although the required sample size was achieved based on a prior power analysis for correlation analysis, a larger cohort would be necessary to conduct more confident multivariate analyses. Third, the study population was limited to female patients aged ≥50 years with MMPRT. While this reflects the typical epidemiological characteristics of MMPRT, the findings may not be generalizable to other populations, such as men, younger patients or individuals without MMPRT. Fourth, during the study period, all patients had a KL grade ≤ 2. As osteoarthritis severity may influence subchondral bone characteristics, these findings should be interpreted as representative of early‐stage osteoarthritis. However, this population aligns with the current indications for MMPRT repair. Fifth, the SIFK evaluation did not include tibial lesions. The association between tibial bone marrow lesions and SIFK may differ, limiting the comprehensive assessment of subchondral lesions. Sixth, this was a single‐centre study, which may have introduced selection bias and limited external validity. Seventh, the SIFK patients included in this study were predominantly classified as Grade 1 or 2. Therefore, it should be noted that similar results may not be applicable to patients with more advanced grades of SIFK. Eighth, MFC BMD was measured using a custom‐defined ROI. Although the measurement demonstrated excellent reliability (ICC ≥ 0.90), a standardized method for local ROI placement has not yet been established, and further refinement of ROI definition may improve the accuracy of regional BMD assessment. Ninth, preoperative and postoperative patient‐reported outcome measures were not available in this study. Therefore, the clinical impact of SIFK and low BMD on patient symptoms and functional outcomes could not be directly evaluated. Despite these limitations, this study provides new insights into the association between MFC BMD and SIFK in patients with MMPRT. Future research should include large‐scale, multicenter cohort studies with more diverse populations.

## CONCLUSION

MFC BMD showed a significant correlation with BMD in commonly assessed regions of the proximal femur and lumbar spine in patients with early‐stage osteoarthritis. Furthermore, MFC BMD was associated with the presence of SIFK in female patients with MMPRT.

## AUTHOR CONTRIBUTIONS

*Conceptualization*: Takayuki Furumatsu and Koki Kawada. *Methodology*: Takayuki Furumatsu and Koki Kawada. *Formal analysis and investigation*: Koki Kawada and Yuta Morinobu. *Writing—original draft preparation*: Takayuki Furumatsu and Koki Kawada. *Writing—review and editing*: Takayuki Furumatsu, Koki Kawada, Kazuhisa Sugiu, Takaaki Hiranaka, Yuta Morinobu and Yuki Okazaki.

## CONFLICT OF INTEREST STATEMENT

The authors declare no conflict of interest.

## FUNDING INFORMATION

The authors have no funding to report.

## ETHICS STATEMENT

This study was performed in line with the principles of the Declaration of Helsinki. Approval was granted by the Ethics Committee of Japanese Red Cross Okayama Hospital (No. 2024‐37). Written informed consent was obtained from all the patients.

## Data Availability

The data that support the findings of this study are available from the corresponding author upon reasonable request.
